# Slower Waning of Anti-SARS-CoV-2 IgG Levels Six Months after the Booster Dose Compared to Primary Vaccination

**DOI:** 10.3390/vaccines10111813

**Published:** 2022-10-28

**Authors:** Sanja Zember, Kristian Bodulić, Nataša Cetinić Balent, Radojka Mikulić, Alemka Markotić, Oktavija Đaković Rode

**Affiliations:** 1 Health Care Quality Unit, University Hospital for Infectious Diseases “Dr. Fran Mihaljević”, 10000 Zagreb, Croatia; 2 Research Department, University Hospital for Infectious Diseases “Dr. Fran Mihaljević”, 10000 Zagreb, Croatia; 3 Clinical Microbiology Department, Division for Virology, University Hospital for Infectious Diseases “Dr. Fran Mihaljević”, 10000 Zagreb, Croatia; 4 School of Medicine, Catholic University of Croatia, 10000 Zagreb, Croatia; 5 Faculty of Medicine, University of Rijeka, 51000 Rijeka, Croatia; 6 School of Dental Medicine, University of Zagreb, 10000 Zagreb, Croatia

**Keywords:** anti-SARS-CoV-2 antibodies, BNT162b2 vaccine, booster dose, healthcare workers, Croatia

## Abstract

Anti-SARS-CoV-2 IgG titer decreases rapidly after primovaccination, leading to a mandatory booster vaccination. We analysed anti-SARS-CoV-2 Spike RBD IgG levels (positive ≥ 50 AU/mL) in 405 healthcare workers (3010 sera) who received a booster dose (BD) 9 months after two-dose BNT162b2 primovaccination. Median antibody titer at the time of BD (582.6 AU/mL) was 1.7-fold and 16.4-fold lower than the peak titer after the first (961.5 AU/mL) and the second vaccine dose (SVD) (10,232.6 AU/mL), respectively. One month after vaccination, IgG titer increased 40.6-fold after BD compared with a 10.8-fold increase after primovaccination. Three months after vaccination, post-booster antibodies decreased significantly slower (2.2-fold) than after primovaccination (3.3-fold). At six months, antibodies decreased slower after BD (4.5-fold; median 5556.0 AU/mL) than after primovaccination (9.6-fold; median 1038.5 AU/mL). Antibody titers before and one month after BD correlated weakly (*r* = 0.30) compared with a strong correlation (*r* = 0.65) between the corresponding post-primovaccination titers. Pre-vaccination COVID-19 had no effect on IgG levels after BD compared with a positive effect after primovaccination. Despite high post-booster IgG levels, 22.5% of participants contracted mild COVID-19. The trend of IgG decline indicates the need for further revaccination, but the vaccine type should be defined according to viral mutations.

## 1. Introduction

Within the first two and a half years of the severe acute respiratory coronavirus type 2 (SARS-CoV-2) pandemic, more than 610 million COVID-19 cases and 6.5 million deaths were reported worldwide [1]. The pandemic of the new coronavirus, which caused a medical, social and economic crisis, initiated the rapid development of vaccines that represent the most effective measure of pandemic control [2]. To date, almost all developed vaccines target the receptor-binding domain (RBD) of the S1 subunit of the SARS-CoV-2 spike protein. Consequently, assessment of the immune response after vaccination is possible using specific serological tests with the SARS-CoV-2 spike protein S1 subunit antigen [3,4].

Anti-SARS-CoV-2 IgG levels are thought to be strongly correlated with neutralizing ability [5,6,7]. Therefore, post-vaccination anti-SARS-CoV-2 IgG level monitoring seems to be useful in assessing vaccine efficacy and revaccination planning [8]. While COVID-19-naïve individuals achieve peak antibody levels after the second vaccine dose (SVD), individuals with a pre-vaccination history of COVID-19 reach the plateau in antibody levels after the first vaccine dose (FVD) [9,10,11,12,13,14]. Antibody levels after vaccination show an exponential decline three and six months after primovaccination [9,10,11,12,14]. However, individuals who contracted COVID-19 before vaccination generally exhibit higher IgG levels and neutralizing activity 3 and 6 months after primovaccination [14,15]. In addition, it has been shown that individuals vaccinated after recovering from COVID-19 exhibit a lower re-infection rate compared to COVID-19-naïve vaccinees, suggesting a beneficial influence of the so-called hybrid immunity [16]. The main role of booster vaccination is to reduce the rate of SARS-CoV-2 infection and the risk of severe forms of COVID-19 [17,18]. A significant rapid increase in IgG titers after the booster vaccination was noted, implying that protection against COVID-19 can be predicted very soon after receiving the booster dose (BD). Post-vaccination antibody levels are usually measured one month after vaccination, with few studies focusing on antibodies in the first weeks after the booster vaccination [19,20,21,22]. Compared to primovaccination, a slower decline in IgG titer was observed 3 and 4 months after the booster vaccination [23,24].

This longitudinal prospective study aimed to evaluate the anti-SARS-CoV-2 IgG dynamics in response to BNT162b2 (BioNTech, Pfizer, Mainz, Germany) booster vaccination at five time points: just before the BD, one week after the BD and 1, 3 and 6 months after the BD. Antibody dynamics were compared between the respective time points after booster vaccination and primovaccination. A special analysis of the impact of COVID-19 on post-booster antibody dynamics was also included. Our findings may be useful in evaluating the immunogenicity of the BNT162b2 booster dose and assessing its protection against COVID-19 at different time points.

## 2. Materials and Methods

After analysing the anti-SARS-CoV-2 IgG dynamics three weeks after the FVD and 1, 3 and 6 months after the completed two-dose BNT162b2 primovaccination [14], we extended the study and monitored the humoral response after the BNT162b2 booster vaccination. Anti-SARS-CoV-2 IgG antibodies were measured just before the BD, i.e., 9 months after the SVD, one week after the BD and 1, 3 and 6 months after the BD. This study included 405 immunocompetent healthcare workers (HCWs) from the University Hospital for Infectious Diseases in Zagreb, Croatia, who were previously monitored at four time points after primovaccination and boosted with the BNT162b2 vaccine nine months after primovaccination. Before receiving the BD, all HCWs were asked to continue participating in antibody monitoring, and those who agreed signed an informed consent. The demographic and COVID-19 history data of participants were collected at defined time points. The diagnosis of COVID-19 was made by RT-PCR and anti-Np SARS-CoV-2 antibody determination. The study was approved by the Institutional Clinical Research Ethics Committee.

Anti-SARS-CoV-2 IgG antibody levels were measured using a quantitative chemiluminescent microparticle immunoassay targeting the receptor-binding domain (RBD) of the SARS-CoV-2 spike S1 subunit protein (CMIA, SARS-CoV-2 IgG II Quant, Architect, Abbott, Chicago, IL, USA). According to manufacturer instructions, the cut-off value for a positive result was 50 AU/mL. The manufacturer declared 100% (95% CI: 95.72–100%) positive agreement with neutralization testing results [14,25].

The association between categorical variables was tested with Fisher’s exact test. Confidence intervals for proportions were calculated using the Agresti–Coull method. Antibody titers at different time points and antibody fold changes were compared with the Wilcoxon signed-rank test. Antibody titers between different participant categories were compared with the Mann–Whitney U test. Pairwise correlations between numerical variables were analysed with Spearman’s correlation coefficient and the correlation test. All tests were two-tailed with the significance level set to 95%. *p*-values were corrected for multiple testing with the Bonferroni method. Statistical analysis and data visualization were performed in R (version 4.1.0.) with ggplot2 (version 2.3.3.) and ggpubr (version 0.4.0.) packages [26].

## 3. Results

### 3.1. Sample and Participant Characteristics

We analysed anti-SARS-CoV-2 IgG dynamics in 3010 consecutive sera from 405 HCWs. Of the 1525 booster-related sera, 392 sera were collected just before the booster vaccination and 1133 sera were collected after BD. A total of 1485 sera collected after primovaccination from the same HCWs was also included. The mean participants’ age was 43.4 years (range 20.1–66.5 years) and 82.0% of participants were female. A total of 45 (11.1%) HCWs were diagnosed with COVID-19 before primovaccination, and 26 (6.4%) HCWs had COVID-19 after primovaccination and before booster vaccination. Furthermore, 91 (22.5%) HCWs contracted COVID-19 after the booster vaccination: 6 (6.6%) one week to one month after BD, 56 (61.5%) between one and three months, and 29 (31.9%) between three and six months after BD (Table 1). Similar post-booster infection rates were noted in HCWs with a history of COVID-19 before primovaccination (10, 24.4%) and COVID-19-naïve HCWs (81, 22.2%, *p* = 0.312). All participants infected with SARS-CoV-2 after booster vaccination were asymptomatic or developed a mild form of the disease. Sera of HCWs diagnosed with COVID-19 after vaccination were excluded from antibody level comparisons, correlation analyses and antibody fold change comparisons.

### 3.2. Anti-SARS-CoV-2 Levels after Primary Vaccination and the Booster Dose

**Figure 1:**
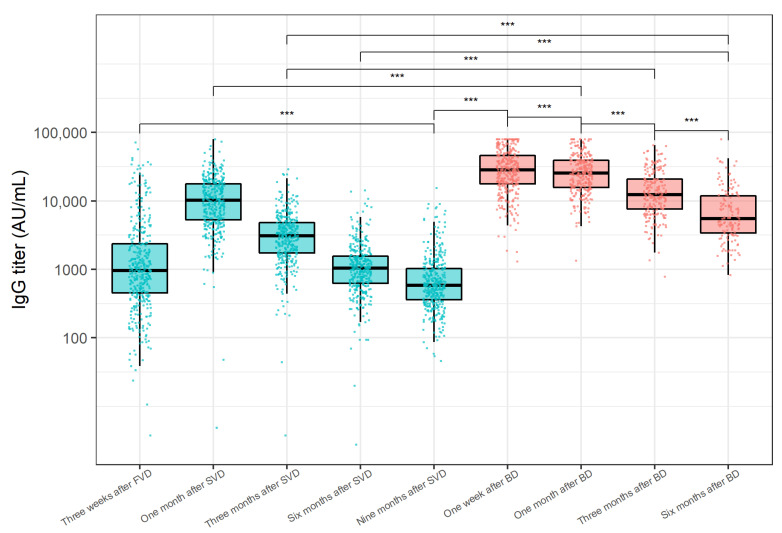
Anti-SARS-CoV-2 IgG titer distributions at analysed time points after BNT162b2 primovaccination (blue) and the booster BNT162b2 vaccination (red). The y-axis is logarithmically scaled. The boxes show the median and interquartile ranges of the distribution, while the whiskers extend to the minimum and maximum nonoutlier values of the distribution. Points denote individual participants. Sera of participants acquired after COVID-19 onset were not included. FVD = first vaccine dose, SVD = second vaccine dose, BD = booster dose. *** *p* < 0.001 (Wilcoxon signed-rank test, Bonferroni adjustment for multiple comparisons).

The distribution of anti-SARS-CoV-2 IgG levels at the analysed time points is shown in Figure 1. A significant increase in anti-SARS-CoV-2 IgG levels was shown one month after the primovaccination, followed by a steady decrease 3 and 6 months after the SVD. The decline in antibody levels continued 9 months after the SVD, resulting in significantly lower antibody levels when compared to antibody levels three weeks after the FVD (medians 582.6 and 961.5 AU/mL, *p* < 0.001). As early as one week after the BD, a substantial increase in IgG antibodies was recorded (median 28,416.0 AU/mL, *p* < 0.001). Interestingly, 64.3% (95% CI 60.0–68.7%) of participants exhibited a significant decline in IgG levels one month after the BD (median 25,633.0 AU/mL) when compared to antibody levels one week after the BD (*p* < 0.001). When comparing the IgG levels one month after vaccination, the antibody titer after booster vaccination was significantly higher than the antibody titer after the primovaccination (*p* < 0.001). Anti-SARS-CoV-2 IgG titer significantly decreased 3 (median 12,406.0 AU/mL, *p* < 0.001) and 6 months (median 5550.6 AU/mL, *p* < 0.001) after the BD. Even 6 months after the BD, antibody levels were significantly higher than antibody levels 3 months after primovaccination (*p* < 0.001).

**Figure 2:**
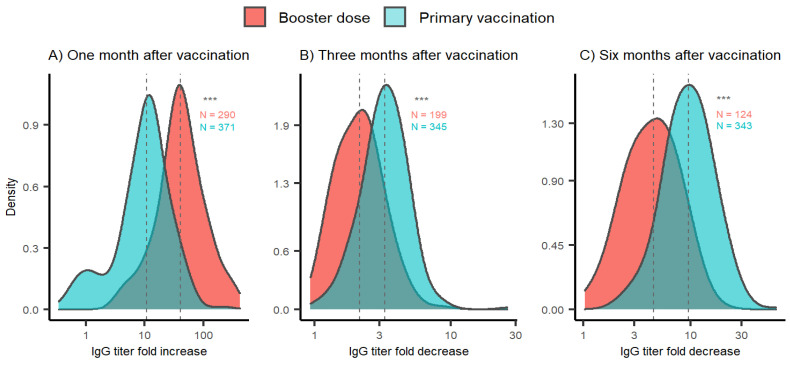
Anti-SARS-CoV-2 titer fold change distribution after primovaccination (blue) and booster vaccination (red). (**A**) Titer fold changes one month after vaccination calculated relative to IgG titers before the respective vaccination. (**B**,**C**) Titer fold changes three and six months after vaccination calculated relative to IgG titer one month after the respective vaccination. Sera of participants acquired after COVID-19 onset were not included. The x-axis is logarithmically scaled to increase resolution. The y-axis represents the Gaussian kernel density estimate of the probability density function. Dotted grey lines denote medians of IgG level fold change distributions. Sample sizes are indicated alongside the respective distribution. *** *p* < 0.001 (Wilcoxon signed-rank test).

In order to further compare the antibody dynamics after primovaccination and the booster vaccination, we analysed the IgG fold changes between the respective time points (Figure 2). One month after the BD, the IgG levels increased 40.6-fold on average, which is significantly higher compared to the increase in IgG titer after primovaccination (median 10.8 times, *p* < 0.001). Three months after the BD, the IgG titer continued to decline (median 2.2-fold). This decline was significantly slower than IgG titer decline recorded after the primovaccination (median 3.3-fold, *p* < 0.001). Furthermore, the fold decrease in IgG titer 6 months after the BD (median 4.5-fold) was significantly lower than the decline in IgG titer 6 months after primovaccination (median 9.6-fold, *p* < 0.001) when compared to IgG levels one month after the respective vaccination.

**Figure 3:**
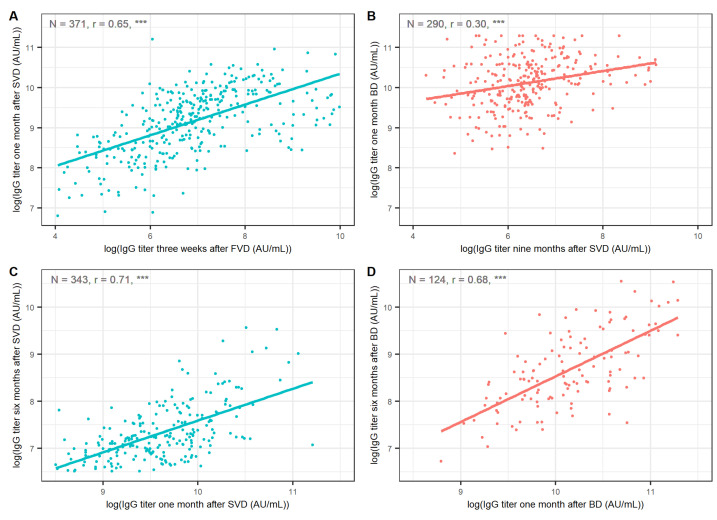
Correlations of antibody titers (**A**) one month after SVD and three weeks after FVD, (**B**) one month after BD and nine months after SVD, (**C**) six months after SVD and one month after SVD, (**D**) six months after BD and one month after BD. Points corresponding to IgG titers after primovaccination are coloured blue; points corresponding to IgG titers after the BD are coloured red. Titers were logarithmically transformed before plotting to ensure better visualisation. Sera after contracting COVID-19 were not included. FVD = first vaccine dose, SVD = second vaccine dose, BD = booster dose, *r* = Spearman’s correlation coefficient. *** *p* < 0.001 (correlation test).

We also compared the correlations between IgG titers at the respective time points after primovaccination and after booster vaccination (Figure 3). Antibody levels three weeks after the FVD exhibited a strong correlation with antibody levels one month after the SVD (*r* = 0.65, 95% CI 0.59–0.71, *p* < 0.001). The correlation between IgG titers just before the BD and one month after the BD was notably weaker (*r* = 0.30, 95% CI 0.19–0.40, *p* < 0.001). Antibody titers one month and six months after the SVD were strongly correlated (*r* = 0.71, 95% CI 0.65–0.75, *p* < 0.001). Similarly, antibody titers one month and six months after the BD exhibited a strong correlation (*r* = 0.68, 95% CI 0.57–0.77, *p* < 0.001).

### 3.3. Anti-SARS-CoV-2 Levels According to History of COVID-19, Sex and Age

The analysis of anti-SARS-CoV-2 IgG levels according to HCWs’ history of COVID-19, sex and age is shown in Table 2. Participants with a history of COVID-19 before vaccination had significantly higher IgG levels than previously uninfected HCWs 3 and 6 months after primovaccination. These HCWs maintained higher IgG levels even 9 months after primovaccination (median 1067.7 vs. 556.0 AU/mL in COVID-19-naïve HCWs, *p* < 0.001). However, HCWs with a pre-vaccination history of COVID-19 did not show significantly higher antibody titers at any of the analysed time points after the BD (*p* > 0.05). We also examined whether participants who acquired COVID-19 after booster vaccination had lower IgG titers at the last time point before COVID-19 onset. A total of 56 participants who contracted COVID-19 between one and three months after booster vaccination did not show significantly lower antibody levels one month after the BD (*p* > 0.05). However, 29 HCWs who had COVID-19 between 3 and 6 months after the BD had significantly lower IgG titers three months after the BD than the other participants (medians 10,219.0 and 12,406.0 AU/mL, *p* = 0.039).

When considering participants’ gender, anti-SARS-CoV-2 IgG titers did not significantly differ between male and female HCWs at any of the examined points (*p* > 0.05). Antibody levels showed a weak negative correlation with age at every time point after primovaccination. This trend continued nine months after the SVD (*r* = −0.23, *p* = 0.003). However, subjects did not show a significant correlation between age and antibody titer at any time points after receiving the BD (*p* > 0.05).

## 4. Discussion

This study is part of a 15-month follow-up on anti-SARS-CoV-2 IgG post-vaccination dynamics, focused on the IgG immune response after one BNT162b2 booster dose. As such, this work represents a continuation of our previous study on IgG levels after primary vaccination with two doses of BNT162b2 vaccine [14]. A significant increase in anti-SARS-CoV-2 IgG was observed as early as one week after the BD, which suggests a strong activation of specific memory B lymphocytes and differentiation of plasma cells within a few days after vaccination. This can be considered a significant discovery in terms of the rapid achievement of strong immune protection after boosting. One month after the BD, most vaccinees exhibited a decrease in IgG levels compared to IgG levels one week after the BD. This result indicates that the peak post-booster humoral immunity is achieved in most cases less than a month after the BD. Notably, the antibody levels one month after the BD were on average 40.6 times higher than the antibody levels before the BD, which is consistent with the results of similar studies [18,19,20,21]. The significantly higher increase in anti-SARS-CoV-2 IgG one month after booster vaccination compared to the increase in IgG levels after primovaccination confirms the strong induction of pre-existing memory B-cells by the BD.

Three months after the BD, antibody levels decreased 2.2-fold on average, which was still significantly slower than the decline in IgG levels 3 months after primovaccination. The same trend continued after 6 months, when a post-booster titer decline of 4.5 times was recorded compared to the IgG level measured one month after booster vaccination. The higher peak antibody levels one week after the BD and the slower decline in IgG compared with primovaccination indicate a beneficial effect of the BNT162b2 booster dose.

The correlation between pre- and post-booster antibody levels was relatively weak, suggesting that post-booster IgG levels cannot be reliably predicted by pre-booster or post-primovaccination IgG levels. This observation contrasts the strong correlation between antibody levels before and after the SVD, which initiates the primary immune response development. However, IgG titers 1 and 6 months after the BD were strongly correlated, which was similar to trends observed after primovaccination [14]. These findings suggest that high levels of antibodies one month after the BD probably correlate with prolonged humoral immunity.

Furthermore, we analysed the impact of pre- and post-vaccination COVID-19 on the humoral immune response after booster vaccination. Contraction of COVID-19 before primovaccination did not affect antibody levels at any of the analysed post-booster time points. This finding confirms the waning of specific antibody levels after vaccination regardless of pre-vaccination COVID-19 history [27,28]. Levels of specific memory B-cells show a similar decline after primovaccination as after COVID-19 [29,30]. This implies that post-vaccination antibody levels in individuals with a history of pre-vaccination COVID-19 could potentially predict IgG levels after the fourth vaccine dose in SARS-CoV-2-naïve individuals. Therefore, IgG levels in individuals who had COVID-19 before vaccination could be indicative of the results to be expected after the fourth vaccine dose.

Studies have shown that vaccinees with a history of COVID-19 prior to vaccination had higher levels of anti-SARS-CoV-2 IgG and a lower rate of reinfection with the Omicron variant than COVID-19-naïve individuals vaccinated with two vaccine doses [14,15,16]. In our study, a relatively large number of HCWs (22.5%) had COVID-19 after booster vaccination. Most of these cases were recorded between one and three months after the BD, which coincided with the new wave of cases in Croatia caused by the Omicron variant [1]. Additionally, 10 participants (22.2%) with a pre-vaccination COVID-19 history developed COVID-19 after booster vaccination. The relatively high post-booster SARS-CoV-2 infection rate can be explained by mutations in the spike antigen, resulting in vaccine-induced antibodies failing to recognize the spike antigen [31,32,33]. The high infection rate could also be a consequence of the depletion of the immune capacity of specific memory B-cells over time [9,10,11,12,13,14,27,28,29,30]. Participants who developed COVID-19 between three and six months after booster vaccination had lower pre-illness anti-SARS-CoV-2 IgG levels. Nevertheless, these antibody levels were still comparable to peak IgG levels after primovaccination. On the other hand, vaccinees who became infected between one and three months did not exhibit lower pre-illness IgG titers. Therefore, drawing conclusions about the protection against infection and effectiveness of immunity based on the level of the post-vaccination titer reached is presumptuous.

Even though previous studies demonstrated a negative correlation between the antibody levels after the primovaccination and older age [9,10,12,14], no significant correlation between age and IgG titer was found after booster vaccination at any of the analysed time points [21,22]. This indicates a high degree of immunogenicity of the BNT162b2 booster dose in all age groups, and emphasizes the importance of administering the BD in older individuals. Finally, the lack of association between post-booster antibody levels and participants’ gender is consistent with trends observed after primovaccination [14,21].

Several limitations of our study should be noted. The participants of this study were HCWs aged 18 to 65, implying that the results of this study refer to the adult population and cannot be extrapolated to children and individuals older than 65 years. Another limitation of the analysed sample was the strong gender imbalance. However, we believe that this did not affect the conclusions of our study given that post-vaccination IgG levels are generally independent of gender [14,21]. It is also necessary to mention the potential problem of defining the participants’ COVID-19 status at the analysed time points. The diagnosis of COVID-19 was made by RT-PCR SARS-CoV-2 findings or according to retrospective anti-Np SARS-CoV-2 antibody tests. Given that the BNT162b2 vaccine targets the recombinant S antigen, NP-specific antibodies represent a valuable tool in the retrospective detection of COVID-19. However, a potential disadvantage of this method is the relatively rapid decline of Np-specific antibodies after the onset of COVID-19. Consequently, some asymptomatic participants could have remained undiagnosed [23,34]. However, the HCWs participating in this study were also monitored clinically, suggesting that the number of presumed undetected cases should be considered minor and insufficient to affect the conclusions of this study.

## 5. Conclusions

A significant increase in anti-SARS-CoV-2 IgG was recorded as early as one week after the booster, resulting in significantly higher peak antibody levels compared to primovaccination. A decrease in anti-SARS-CoV-2 IgG level was already noted one month after the booster vaccination and continued for 6 months, but was significantly slower than the IgG antibody decrease after primovaccination. The correlation between pre- and post-booster antibody levels was weak. Pre-vaccination COVID-19 had no effect on IgG levels after the booster. Despite the strong humoral immune response induced by the booster dose, a significant number of participants contracted post-booster COVID-19 as a result of infection with the Omicron variant and, possibly, antibody waning. However, all of these HCWs presented with a mild or asymptomatic form of the disease. Therefore, the importance of vaccination and the need for boosters as an effective protection measure against severe forms of COVID-19 are unquestionable. Even after the booster, antibody levels decrease quickly, which necessitates revaccination. However, the vaccine should be adapted to current variants of circulating viruses.

## Figures and Tables

**Table 1:** Serum samples and acquired COVID-19 after vaccination by testing time point.

Testing Time Point	Serum Samples *N*	COVID-19 *n* (%)
Three weeks after FVD	388	-
One month after SVD	375	0
Three months after SVD	359	6 (1.7%)
Six months after SVD	363	4 (1.1%)
Nine months after SVD	392	16 (4.1%)
One week after BD	344	0
One month after BD	301	6 (2.0%)
Three months after BD	275	56 (20.4%)
Six months after BD	213	29 (13.6%)
Total	3010	117 (3.9%)

FVD = first vaccine dose, SVD = second vaccine dose, BD = booster dose.

**Table 2:** Anti-SARS-CoV-2 IgG titer after BNT162b2 primovaccination and booster vaccination according to history of COVID-19, sex and age.

Anti-SARS-CoV-2 IgG AU/mL Median (IQR)							Correlation Analysis	
Time Point	Pre-Vaccination COVID-19			Sex			Age	
	Yes (*N* = 45)	No (*N* = 364)	*p*	Male (*N* = 73)	Female (*N* = 332)	*p*	*r*	*p*
Three weeks after FVD * (*N* = 388)	13,460.0 (7730.0–22,167.0)	832.4 (400.0–1560.1)	<0.001	907.4 (461.2–2390.2)	965.5 (461.2–2390.2)	0.284	−0.42	<0.001
One month after SVD * (*N* = 375)	11,085.0 (8380.0–19,718.0)	9997.1 (5024.7–17,318.4)	0.065	8054.0 (3850.0–18,979.0)	10,416.4 (5786.5–17,415.7)	0.132	−0.19	0.006
Three months after SVD (*N* = 353)	4049.0 (2524.0–6018.0)	3004.9 (1672.0–4652.1)	0.007	2823.7 (1330.2–4878.6)	3176.2 (1878.5–4827.8)	0.398	−0.20	0.005
Six months after SVD (*N* = 353)	1443.9 (1006.8–3416.0)	1000.8 (587.9–1493.9)	<0.001	936.5 (504.8–1650.2)	1058.0 (655.6–1547.2)	0.532	−0.22	0.004
Nine months after SVD (*N* = 370)	1067.7 (583.3–2425.4)	556.0 (336.5–878.4)	<0.001	598.0 (361.8–1026.8)	578.1 (361.8–1026.8)	0.912	−0.23	0.003
One week after BD * (*N* = 323)	21,094.0 (16,772.0–35,419.0)	28,887.0 (18,072.0–48,255.0)	0.072	30,627.0 (13,856.0–44,039.0)	27,868.0 (17,747.0–45,866.0)	0.671	−0.11	0.080
One month after BD (*N* = 279)	18,959.0 (11,437.0–27,895.0)	26,232.0 (16,631.0–39,262.0)	0.094	24,469.0 (17,215.0–35,537.0)	25,734.0 (15,329.0–39,147.0)	0.714	0.14	0.061
Three months after BD (*N* = 209)	10,832.0 (5492.0–23,757.0)	12,569.0 (8008.0–20,779.0)	0.292	12,842.0 (8102.0–24,705.0)	12,406.0 (7562.0–20,845.0)	0.644	0.19	0.064
Six months after BD (*N* = 139)	4356.0 (2716.0–8386.0)	5609.9 (3603.1–12,056.4)	0.321	6887.0 (4354.0–13,903.0)	5040.2 (3257.2–10,960.3)	0.194	0.13	0.052

* FVD = first vaccine dose, SVD = second vaccine dose, BD = booster dose, IQR = interquartile range, ^#^ *r* = Spearman correlation coefficient. Sera from participants after COVID-19 diagnosis were not included. *p*-values were corrected for multiple testing with the Bonferroni method.

## Data Availability

The data presented in this study are available on request from the corresponding author. The data are not publicly available due to privacy and ethical restrictions.
